# Dietary Intake and Supplement Use Among Saudi Residents during COVID-19 Lockdown

**DOI:** 10.3390/ijerph18126435

**Published:** 2021-06-14

**Authors:** Hanan A. Alfawaz, Nasiruddin Khan, Ghadah A. Aljumah, Syed D. Hussain, Nasser M. Al-Daghri

**Affiliations:** 1 Department of Food Science & Nutrition, College of Food & Agriculture Science, King Saud University, Riyadh 11495, Saudi Arabia; halfawaz@ksu.edu.sa; 2 Biomarkers of Chronic Diseases, Biochemistry Department, King Saud University, Riyadh 11451, Saudi Arabia; danishhussain121@gmail.com; 3 Department of Food Science and Human Nutrition, College of Applied and Health Sciences, A’ Sharqiyah University, Ibra 400, Oman; knasiruddin@asu.edu.om; 4 King Abdulaziz Medical City, Riyadh 14611, Saudi Arabia; Aljumahgh@ngha.med.sa

**Keywords:** diet behavior, dietary supplements, COVID-19, income, education, multivitamin, Saudi Arabia

## Abstract

Healthy diet and supplement use may prove as sustainable strategies to lower COVID-19 infection. Our study investigated the dietary changes before and during lockdown and observed dietary supplements (DS) use among residents in Saudi Arabia. This cross-sectional study collected data via an online electronic survey questionnaire among males (N = 921) and females (N = 1044) residing in Saudi Arabia, 15 years of age and above. There was a significant decrease in the prevalence of males (before vs. during lockdown) having improved changes in dietary habit (68.6% vs. 65.8%; *p* = 0.004), which was similar in female participants (69 vs. 73.4% vs. 69%; *p* < 0.001). The frequency of multivitamin users among COVID-19 participants was significantly lower than non-users (44.4 vs. 55.6; *p* < 0.003). Male respondents within 26–35 years of age were more likely to use multivitamin supplements than females (30.1 vs. 22.6%; *p* < 0.05) of same age group. Predictors for DS use were increased age group, income, education level and COVID-19 status. In conclusion, an increase in unhealthy diet behavior was observed among Saudi males and females during the pandemic lockdown and the predictors of DS use included increased age, income, education level and COVID-19 status.

## 1. Introduction

The coronavirus disease 2019 (COVID-19) was declared as global pandemic on 11 March 2020 by World Health Organization (WHO). The disease cost innumerable human lives and infected millions worldwide. At present, several COVID-19 vaccines have been approved for human use that have not been rolled out yet worldwide, due to challenges such as its surplus production, reasonable price, balanced distribution to all needy countries and delivery at the local level [1]. Moreover, to make the situation worse, high rates of vaccine hesitancy were reported from countries like in Jordan, Kuwait and Saudi Arabia [2], and in Europe, the rates of COVID-19 vaccine hesitancy were 41% in Italy and 26% in France [3,4]. These issues could pose a serious threat to the preventive measures aimed at controlling COVID-19 spread at the global level. Hence, in this situation, following preventive measures and strategies such as having a balanced diet may prove extremely beneficial, since the link between viral infection, nutrition and immune system, are well established [5].

Home confinement during COVID-19 impacted negatively on diet quality among adolescents from various countries such as Italy, Spain, Chile, Colombia and Brazil [6]. A recent report from Saudi Arabia demonstrated unhealthy changes in dietary habits, compromising the quality and quantity of the food [7]. The lockdown is a compulsory step to limit the rapid COVID-19 infection among individuals, however, this step has negative consequences especially related to diet and personal health. The confinement period is accompanied with reduced food accessibility and availability, which in turn compromises diet quality. Unhealthy dietary habits and lifestyle behavior including consumption of more food [8], snacking, decrease in sports activity, additional weight gain, psychological stress leading to change in dietary pattern have been reported recently [9,10,11]. Moreover, studies during COVID-19 lockdown reported a higher degree of emotional eating during the lockdown (e.g., sweets, pastries and alcohol), poor quality sleep, increase in the amount of screen time (for fun and entertainment), and sedentary behaviors [12,13,14].

For the last few decades, the use of dietary supplements has increased all over the world, with its consumption based on age, gender, illness prevention and nutritional deficiencies [15]. Based on European Food Safety Authority (EFSA), dietary supplements (DS) are defined as “concentrated sources of nutrients or other substances with a nutritional or physiological effect intended to supplement a normal diet” [16]. Recent studies from Saudi Arabia have shown the increased prevalence of DS use among its population. However, these studies recommend the consumers to use it with a doctor’s prescription and advocates for increased awareness about its health effects [17,18]. Studies have demonstrated the positive health effects of functional foods on respiratory, viral and parasitic, infectious, and inflammatory diseases [19]. As the COVID-19 pandemic is thought to be related to the immune system, the individuals are paying attention towards improving their diet in order to boost their immunity as a strategy to curb COVID-19 infection. Recently, the information in various social-media platforms and advertisements favoring the role of DS use to fight against COVID-19 prompted the consumer all around the world to increase its use [20,21].

Overall, the individual dietary habits and lifestyle behaviors are highly affected during this lockdown, leading them to acquire possible ways to combat against infection which may either prove beneficial or detrimental in terms of future health. Furthermore, since the lockdown coincided with the holy month of Ramadan, it is important to know how Ramadan observers in the era of COVID-19 changed their dietary behaviors in response to both the pandemic and the call for intermittent fasting, knowing that in theory, periodic fasting enhances the immune system [22]. It is therefore essential to study the extent of variation in dietary habits and observe the frequency of supplement use during the current pandemic to ascertain sustainable ways as a strategy to fight against rising infection. Hence, the aim of our present study is to observe dietary habit before and after lockdown period and demonstrate the DS use and its predictors among adult Saudi and non-Saudi population.

## 2. Materials and Methods

### 2.1. Study Design and Participants

This cross-sectional survey was conducted from 11 May to 6 June 2020, corresponding to almost 2 weeks during and after Ramadan (23 April–23 May 2020), using an online electronic survey. Within this timeframe, confirmed COVID-19 cases in Saudi Arabia increased from 41,014 to 98,869 [23]. Table 1 shows the timeline of events with respect to restrictions imposed in Saudi Arabia [24]. For the purpose of the study, the ‘lockdown’ refers to the curfews imposed. All adult Saudi citizens and residents (non-Saudis) 18-years-old and above with access to the internet were deemed eligible to voluntarily participate in the survey. All participants were asked for their email addresses for verification purposes only and to ensure that each participant completed the survey only once. The study design and protocol were approved by the Ethics Committee for Scientific Research and Post Graduate Studies at the College of Science, King Saud University, Saudi Arabia (reference# KSU-HE-20-246). The survey had no risks other than potential inconvenience during participation. 

### 2.2. Questionnaire

The questionnaire included a cover letter in Arabic and English. It consisted of demographic and social information, and statements in Likert scale format to determine changes in dietary habits before and during lockdown. Participants have to indicate their habits before and during lockdown for the same question. Experts in the related field reviewed the questionnaire and several revisions were made to strengthen the reliability and enhance scientific value of the data to be collected. A pilot study (N = 75 participants) was performed to confirm reliability and validity of the questionnaire and obtained Cronbach’s α, which was noted to be excellent (84%). After completion, the questionnaire was transferred to an online link for distribution to different social media outlets throughout Saudi Arabia.

The questionnaire consisted of following sections:

(1) Anthropometry and Sociodemographic characteristics, including age, sex, marital status, nationality, family income, family members, educational qualification, employment status, and region. The family income was categorized depending on the amount of Saudi Arabian Riyal (1 SAR = 0.266 USD)
(2) The dietary habit and behavior questionnaire before and during the pandemic lockdown based on Likert scale statement among all, male and female participants.
(3) Supplements used during the quarantine, including dietary supplements containing vitamin D, selenium, zinc, and vitamin C based on gender, COVID-19 status, education, income, age, and nationality.

### 2.3. Sample Size Calculation

Based on the prevalence of supplement use in Saudi population reported in the literature, i.e., 22% [25], the minimum required sample size at 95% CI with 2% precision was 1648. To overcome the expected non-response rate of 20%, a questionnaire was sent to 1965 potential participants. 

### 2.4. Data Analysis

Analysis was done using SPSS version 16.5 (SPSS Inc., Chicago, IL, USA). Continuous variables were presented as mean ± standard deviation while categorical variables were presented as frequencies (N) and percentages (%). The chi-Square test was used to determine differences between categorical variables of interest. The independent *t*-test was used to determine differences between normal continuous variables. The McNemar test was used to examine differences in dietary habits before and during lockdown. To examine the predictors of DS use, binomial logistic regression analysis was performed (Odds Ratios and 95% CI) with supplementation as dependent and socio-demographics as independent variables. The *p*-value was considered significant at <0.05.

## 3. Results

### 3.1. Demographic Characterstics of the Participants

Table 2 represents demographic characteristic of the participants that have also been published in our previous paper [24]. The frequency of married male participants was significantly higher than females (61.6 vs. 45.4%, *p* < 0.001, respectively). 

### 3.2. Dietary Pattern before and during Lockdown

The dietary habit pattern among participants as a whole (before vs. during lockdown) is presented in Table 3. A significantly low proportion of participants agree (combined proportion of strongly agree and agree) that they changed their diet for better health during lockdown than before (67.5 vs. 71.2%, *p* < 0.001). No significant differences were found with regards to statements that during the pandemic: they began to care more about healthy diet (71.4 vs. 71.6%, *p* > 0.05), they reduced their fast-food consumption (77.2 vs. 79%, *p* > 0.05), they have not eaten from any restaurant (73.8 vs. 75.3%, *p* > 0.05), and a higher proportion disagree for increased consumption of homemade food (47.8 vs. 44.9%, *p* > 0.05) than before. However, a significant proportion of participants avoided drinking coffee from coffee shop during lockdown as compared to before (41.2 vs. 40%, *p* = 0.040). 

The dietary habit pattern among males (before vs. during lockdown) exhibited a significant low proportion of male participants agree (combined proportion of strongly agree and agree) that during lockdown: there are better changes in their dietary habit during lockdown (65.8 vs. 68.6%, *p* = 0.004). However, no significant differences were found with regards to the statements: that they reduced their fast-food consumption (78.8 vs. 80.3%, *p* > 0.05), they have not eaten from any restaurant (73.5 vs. 75.1%, *p* > 0.05), and they ate more fresh fruits and vegetables (64.7 vs. 66.8%, *p* > 0.05), but high proportion disagree, although insignificant, about increased consumption of homemade food (46.4 vs. 42.2%, *p* > 0.05), than before. Similarly, the dietary behavior among females (before and during lockdown) demonstrated a significant low proportion of female participants agree (combined proportion of strongly agree and agree) that during the pandemic: there are better changes in their dietary habit (69 vs. 73.4%, *p* = 0.002). However, no significant differences were found with regards to the statements that: they began to care more about healthy diet (69.5 vs. 71.1%, *p* > 0.05), they reduced their fast-food consumption (75.9 vs. 78%, *p* > 0.05), they have not eaten from any restaurant (74 vs. 75.5%, *p* > 0.05), but high proportion disagree about increased consumption of homemade food (49.2 vs. 47.3%, *p* > 0.05), than before. Furthermore, a high frequency of females avoided drinking coffee from coffee shop during lockdown as compared to before (41.9 vs. 39.8%, *p* < 0.035). 

### 3.3. Dietary Supplement Use among Participants 

The overall use of supplements among participants based on gender and among COVID-19 patients and their family members is shown in Table S1. The frequency of various supplement (multivitamin, vitamin D, selenium, and zinc) use were non-significantly high among males than females (26 vs. 21.5%; 21.5 vs. 19.7%; 0.7 vs. 0.3%; 1.4 vs. 1.3%), respectively. Similarly, the overall use of multivitamin and zinc were non-significantly high in COVID-19 males than females (47.4 vs. 42.3), respectively, whereas, use of vitamin D and C were high in females than males (26.9 vs. 10.5; 19.2 vs. 10.5), respectively. Table S2 represents the frequency of supplement use based on COVID status. Among participants diagnosed with COVID-19, the frequency of multivitamin users was significantly low as compared to non-users (44.4 vs. 55.6; *p* < 0.003). No other significant difference was observed for supplement use among non-COVID-19 participants and their family members. Table S3 exhibited gender difference for supplement use among participants based on their education, income an age. The male participants specifically between 26–35 years of age showed significant higher use of multivitamin than females (30.1 vs. 22.6%; *p* < 0.054) of the same age group. Table S4 represents the DS use based on nationality and gender. No significant difference was observed among males and females for DS use in Saudi and non-Saudi nationals.

### 3.4. Predictors of DS Use

Table 4 represents the results of logistic regression outcomes demonstrating the independent predictors of supplement use among participants according to socio-demographic and COVID-status. The significant strong predictors were increased age group, high income and education level and positive COVID-19 status. The multivitamin users were less likely to belong younger age group (15–25 years) (OR: 0.6, 95% CI: 0.5–0.8) as compared to higher age groups. The participants between 15–25 years of age were less likely to be Vitamin D users than high age groups, but the 95% CI included 1. The odds of vitamin C use were 2-fold (95% CI: 1.3–3.2) higher among participants with high income level than low income. Compared to participants with higher education (PhD), those with low educational (bachelor) had a significant lower probability of using vitamin D (OR: 0.6, 95% CI: 0.5–0.9). The probability of multivitamin use was 2.5-fold (95% CI: 1.4–4.5) higher among participants with positive COVID-19 status as then negative status.

## 4. Discussion

The COVID-19 pandemic had a huge impact on human diet and health among all sections of population worldwide. The present study observed an unhealthy change in dietary habit and behavior during the pandemic among all participants irrespective of gender and demonstrated non-significant increase in DS use among males than females. The strong determinants of DS use among participants were increased age, high income and education level and positive COVID-19 status.

### 4.1. Dietary Habit and Behavior

The dietary habits among participants demonstrated an increase in unhealthy diet behavior during lockdown, including less care about their health, increased fast-food consumption, low consumption of fresh fruits and vegetables, and lower preference to homemade food, as compared to before pandemic. The unhealthy change in diet pattern during the pandemic and lockdown period have been reported from around the world, including middle east countries. Study from United Arab Emirates revealed negative lifestyle changes, unbalanced food choices, and psychological problems among individuals during the COVID-19 pandemic [14]. An Italian survey (aged between 12 and 86 years) demonstrated slight weight gain among 48.6% of its population while 37.4% and 35.8% of the study population responded to eat more or less healthy food (fruit, vegetables, nuts and legumes), respectively. Although, the study pointed some healthy behavior change among participants (3.3%) who quit smoking during COVID-19 emergency, most of the population responded not to have changed its habits (46.1%), while 37.2% made them worse [26]. 

With some favorable changes, such as increase in the consumption of homemade food, the Saudi adults had been shown to compromise the quality and quantity of their diet during the pandemic [7]. The COVID-19 pandemic lockdown significantly affected eating habits in German young adults with one-third of study subjects eating more (31.2%), while (16.8%) ate less compared to the time before lockdown [8]. The “Effects of home Confinement on multiple Lifestyle Behaviours during the COVID-19 outbreak (ECLB-COVID19)” Online Survey demonstrated unhealthy eating behavior during lockdown period with increased consumption of unhealthy food, uncontrolled eating and snacking between meals [27]. Our result corroborates the above findings [8,19,20,26,27] showing majority of our participants worsen their dietary habits during lockdown including all, male and female (67.5, 65.8, and 69%), respectively.

The unhealthy dietary habits are more prevalent in Saudi adult population specifically in females than males [28,29]. Our present study agrees with these findings showing overall less interest towards their healthy diet (71.4%), while females exhibited significant less attention towards their healthy diet during the pandemic as compared to before (69.5 vs. 71.1%). A recent polish study demonstrated increased consumption of homemade meals among ProHealth individuals as compared to unhealthy dietary pattern shown by respondents with decrease in homemade meal consumption [30]. On the contrary to Saudi study [7], the present study showed participants with decreased consumption of home cooked meals during lockdown. The results support the above study [30] indicating low consumption of homemade food as one of the factors responsible for overall unhealthy diet behavior during the pandemic lockdown. Moreover, we assume participant’s lack of time and busy lifestyle as a barrier to spent considerable time on healthy meal preparation with easy options such as fast-food consumption and food from restaurant or quick home delivery from other sources. Although the consumption of fruits and vegetables in Saudi adolescent is reported to be far less than recommended [31], its consumption is higher in males as compared to females [32]. However, in contrast to above trend, the present study exhibited significant low consumption of fresh fruits and vegetables in males during the pandemic lockdown than before (64.7 vs. 66.8%). The reason seems to be limited grocery shopping from supermarkets or malls due to their closures, increased online shopping, increased fast-food consumption and paying less attention to healthy food choices. The shutdown and restriction of coffee shops, and restaurants during the pandemic favored an increase in food delivery services. Although considered safe to aid in social distancing, reports suggest that this food distribution approach has a great potential of spreading COVID-19 infection [33,34].

A study from Kuwait revealed no remarkable changes in beverage consumption except for decrease in Americano coffee and fresh juice, possibly due to the closure of coffee shops as a precautionary measure during the pandemic [13]. Our present study supports the above findings [13], demonstrating low coffee consumption specifically in females, possibly due to closure of shops as well as fear of getting contaminated beverage or infection from food delivery services [32,33] during COVID-19 lockdown as compared to before.

### 4.2. Dietary Supplement Use 

The possible role of dietary supplement (such as vitamin C, vitamin D, omega 3 polyunsaturated fatty acids, probiotics, and zinc) as therapeutic agent to fight against COVID-19 infection are under clinical investigation and still not recommended for COVID-19 prevention and treatment [20]. Based on the Council for Responsible Nutrition (CRN)-funded COVID-19 survey (31 July 2020 to 4 August 2020) in US, 91% of DS consumers who increased their supplementation usage was during COVID-19 pandemic, with males consuming more DS than females (47 vs. 39%) with more users aged 18–34 (at 65%). DS consumers increased the usage of specific supplements such as multivitamins, vitamin C, and vitamin D, with usage growing 59%, 44%, and 37%, respectively [35]. The major reasons for increased DS usage were to improve immune strength, and health/wellness with specific augmented use of multivitamin (59%), vitamin C (44%), and vitamin D (37%) [36]. Based on gender, our present results are in agreement with the above survey showing overall increased DS use (multivitamin, vitamin D, selenium, and zinc) among males than females. In case of hospitalized COVID-19 patients, nutritional strategies are thought to improve the condition, as malnutrition is a risk factor to COVID-19 patients [37]. Therefore, DS use (vitamin C, vitamin D, and zinc) may be an effective method to maintain adequate dietary intake for optimal immune function.

Some of the nutrients, vitamins (A, C, D, E, B6, B12, and B9), and minerals (selenium, magnesium, zinc, and copper) are known to play important roles to strengthen our immune systems [38] and so might impact on the treatment of COVID-19.Internet information about methods of combating COVID-19 infection including the use of DSs, differ greatly depending on authenticity of the website (e.g., governmental or commercial) and sometimes compromises quality or may even provide potentially harmful information [39]. A recent study from Saudi Arabia reported that most of the information for the herbal products and DS use were obtained from social media and internet. Moreover, the consumers make their own choice to continue or stop its use based on their personal awareness, knowledge and health condition. In addition, vitamin C was the most commonly used food supplement to reduce the chance of contracting COVID-19 [40]. In our present study, males with positive COVID-19 status showed non-significantly high use of multivitamin and zinc than females, while female COVID-19 patient used more vitamin D and C than males. Moreover, among participants with COVID-19 status, the percentage of multivitamin users was significantly low than non-users. Based on above discussion, there is no plausible reason for these uncertainties in percentage of various DS use based on gender and COVID-19 status. However, our results indicate that participants are choosing and stopping the DS use at their own will without any scientific evidence or recommendation about the role of DS use to treat or prevent COVID-19. In addition, our results supported the abovementioned US survey [35] showing male participants of age (26–35 years) using more multivitamin than females of the same age.

### 4.3. Predictors of DS Use

The strong predictors of DS use among our participants were increased age group and high income. As discussed above, the majority of the multivitamin users in our present study belong to 26–25 years of age group. A recent study about the prospective association between habitual use of vitamin D supplements and risk of COVID-19 infection demonstrated that habitual users of vitamin D supplements were elder as compared to non-users [41]. Our present study corroborates the above findings showing young participants between 15–25 years of age as less likely to be vitamin D users than other higher age groups. The more common use of vitamin D supplements among older adults may prove beneficial in this population, as vitamin D deficiency, as well as diabetes in the Saudi population have proven to be significant risk factors for COVID-19 mortality [42,43,44,45].

Although not significant and uniform, the overall use of various DS was higher among those with high education level. For instance, the overall (positive response among males and females) multivitamin use was high among those having master’s level than bachelor and high school (56.6, 47,2, and 46.2), respectively. Similarly, the overall vitamin D and zinc use was highest (53.4, 5.2%, respectively), among participants with PhD level than other lower educational levels. A similar non-significant pattern was obtained among DS users based on income. The overall multivitamin and vitamin D use was high among those with >5000 SAR than <5000 SAR. High education level had been shown to have a direct association with use of DS [46] Moreover, DS and multivitamin use was shown to be significantly higher among those with a high household income [47].

Our present findings supported the above studies showing increased DS use within the high-income (>5000–7000 SAR and 8000–16,000 SRA) range as compared to low-income level (<5000 SAR). Similarly, the probability of DS use was higher among participants with higher-education levels (PhD) than bachelor degree. As it is clear from Table 4, COVID-19 status is a strong determinant showing increased multivitamin use among those who are COVID-19 positive than COVID-19 negative (44.4 vs. 24.5), respectively.

## 5. Strength and Limitations

To the best of our knowledge, this is the first study in Saudi Arabia that investigated dietary habits and the use of various supplements among both Saudi and non-Saudi population during COVID-19. The strength of our study includes the large sample size during this critical period and reaching the target population in different geographical locations. However, there are a few limitations that need to be considered while interpreting the results of the present study. Due to the cross-sectional nature of the study design itself, we are not able to identify exact causality between the study variables. Moreover, there are limited studies that have evaluated DS’s use based on gender and COVID-19 status, limiting our ability to compare our present findings with other studies. Our results demonstrate a limited number of DS use predictors, with the possibility of missing some important factors of DS use to be taken into account. It is possible that an online survey might not reach some of the target population, due to their location or internet unavailability.

Despite these limitations, the findings of the current study provide the evidence and direction for future research and recommends health organizations to focus on disseminating nutrition awareness about healthy diet behavior and quality food choices as sustainable dietary alternatives, specifically during current pandemic situations. As far as DS use is concerned, the consumers need effective education about authentic websites from which to obtain correct information, and to develop awareness and understanding of both the beneficial and detrimental effects of DS use in COVID-19 treatment and infection. Further investigations and clinical trials are required to establish the role of DS in prevention of COVID-19 infection, and to determine whether DS’s exhibit any real therapeutic value against this pandemic disease. 

## 6. Conclusions

The findings of our study demonstrated a negative impact of COVID-19 lockdown on dietary habit and behavior, with the majority of male and female participants following unhealthier patterns than before. The participants responded that they cared less about a healthy diet, increased their fast-food consumption, and reduced consumption of homemade food and fresh fruit and vegetables during lockdown compared to before. The prevalence of DS use was higher among males than females. Male participants with COVID-19 status used more multivitamin and zinc, while females with positive COVID-19 status used more vitamin D and C. The significant strong predictors of DS use were increased by age group, high income and education level and positive COVID-19 status.

## Figures and Tables

**Table 1:** Timeline of Events [24].

Event	Dates	Curfew Hours	Exceptions
Suspension of non-essential work	15 March		None
Nationwide curfew	23 March–5 April	6 a.m.–7 p.m.	Makkah & Madinah (3 April)
Enhanced curfew	6–25 April	6 a.m.–3 p.m.	None
Ramadan *	26 April–22 May	9 a.m.–5 p.m.	Makkah
Eid Al Fitr *	23–27 May	24 h curfew	None
Phase 1 Partial Easing *	28–30 May	6 a.m.–3 p.m.	Makkah
Phase 2 Partial Easing *	31 May–20 June	6 a.m.–8 p.m.	Makkah

Note: * denotes events that unfolded during the study period (11 May–6 June 2020).

**Table 2:** Anthropometric and socio-demographic information of the participants.

Parameters	All	Males	Females	*p*-Value
N	1965	921 (46.9)	1044 (53.1)	
Marital Status				
Single Married Divorce Widow	860 (43.8) 1040 (52.9) 49 (2.5) 12 (0.6)	355 (38.5) 566 (61.6) 0 (0.0) 0 (0.0)	509 (48.8) 474 (45.4) 49 (4.7) 12 (1.1)	<0.001
Nationality				
Saudi Non Saudi	1632 (83.1) 333 (16.9)	777 (84.4) 144 (15.6)	855 (81.9) 189 (18.1)	0.146
Education				
High School Bachelor Master PHD	165 (8.4) 1158 (58.9) 368 (18.7) 274 (13.9)	75 (8.1) 556 (60.4) 158 (17.2) 132 (14.3)	90 (8.6) 602 (57.7) 210 (20.1) 142 (13.6)	0.359
Family Monthly Income (Valid)				
<5000 5000–7000 8000–16,000 >16,000	687 (35.0) 200 (10.2) 580 (29.5) 498 (25.3)	338 (36.7) 93 (10.1) 258 (28.0) 232 (25.2)	349 (33.4) 107 (10.2) 322 (30.8) 266 (25.5)	0.415
Employment Status				
Employed Unemployed Student Own Business Farmer	1100 (56.0) 217 (11.0) 597 (30.4) 2 (0.1) 49 (2.5)	495 (53.7) 113 (12.3) 286 (31.1) 1 (0.1) 26 (2.8)	605 (58.0) 104 (10.0) 311 (29.8) 1 (0.1) 23 (2.2)	0.295
Family Members				
2–4 4–6 >6 Single	545 (27.7) 700 (35.6) 587 (29.9) 133 (6.8)	261 (28.3) 319 (34.6) 276 (30.0) 65 (7.1)	284 (27.2) 381 (36.5) 311 (29.8) 68 (6.5)	0.820
Age (Year)(Min-Max)	35.2 ± 13.1 (15–75)	35.2 ± 13.1 (16–72)	35.1 ± 12.9 (15–70)	0.799
15–25 year 26–35 year 36–45 year >45 year	609 (31.0) 516 (26.3) 411 (20.9) 429 (21.8)	292 (31.7) 231 (25.1) 187 (20.3) 211 (22.9)	317 (30.4) 285 (27.3) 224 (21.5) 218 (20.9)	0.488
Region				
Riyadh Al Jawf Al Qassim Asir Eastrn Province Hail Jazan Makka Madinah Najran Northern border	1629 (82.9) 7 (0.4) 8 (0.4) 5 (0.3) 81 (4.1) 4 (0.2) 7 (0.4) 72 (3.7) 27 (1.4) 2 (0.1) 11 (0.6)	762 (82.7) 6 (0.7) 0 (0.0) 0 (0.0) 37 (4.0) 0 (0.0) 2 (0.2) 31 (3.4) 14 (1.5) 1 (0.1) 6 (0.7)	867 (83.0) 1 (0.1) 8 (0.8) 5 (0.5) 44 (4.2) 4 (0.4) 5 (0.5) 41 (3.9) 13 (1.2) 1 (0.1) 5 (0.5)	

Note: data presented as N (%). The *p*-values are obtained from the Pearson chi-square test.

**Table 3:** Dietary habit and behavior response before and during lockdown among participants (overall and gender based).

	Overall Participants				Males			Females		
Parameters	Response	Before N (%)	During (%)	*p*-Value	Before N (%)	During (%)	*p*-Value	Before N (%)	During (%)	*p*-Value
After COVID 19, my dietary habits changed for the better	Disagree	233 (11.8)	296 (15.1)	<0.001	115 (12.5)	139 (15.1)	0.004	118 (11.3)	157 (15.0)	0.002
	Agree	1399 (71.2)	1326 (67.5)		632 (68.6)	606 (65.8)		767 (73.4)	720 (69.0)	
I began to care more about healthy diet	Disagree	246 (12.5)	230 (11.7)	0.752	104 (11.3)	91 (9.8)	0.360	142 (13.6)	139 (13.4)	0.771
	Agree	1407 (71.6)	1403 (71.4)		665 (72.2)	677 (73.6)		742 (71.1)	726 (69.5)	
Reduced my fast-food consumption	Disagree	149 (7.6)	182 (9.3)	0.200	65 (7.1)	84 (9.2)	0.362	84 (8.0)	98 (9.4)	0.473
	Agree	1553 (79.0)	1518 (77.2)		739 (80.3)	726 (78.8)		814 (78.0)	792 (75.9)	
Have not eaten from any restaurant during the pandemic	Disagree	184 (9.3)	197 (10.0)	0.497	88 (9.5)	91 (9.9)	1.000	96 (9.2)	106 (10.2)	0.473
	Agree	1480 (75.3)	1450 (73.8)		692 (75.1)	677 (73.5)		788 (75.5)	773 (74.0)	
Avoided drinking coffee from coffee shop	Disagree	784 (39.9)	723 (36.8)	0.040	353 (38.3)	339 (36.8)	0.510	431 (41.2)	384 (36.7)	0.035
	Agree	786 (40.0)	810 (41.2)		370 (40.2)	373 (40.5)		416 (39.8)	437 (41.9)	
Increased consumption of food homemade	Disagree	882 (44.9)	941 (47.8)	0.801	389 (42.2)	427 (46.4)	0.377	493 (47.3)	514 (49.2)	0.720
	Agree	660 (33.6)	621 (31.6)		319 (34.7)	297 (32.2)		341 (32.6)	324 (31.0)	
Ate more fresh fruits and vegetables	Disagree	278 (14.2)	296 (15.0)	0.640	115 (12.5)	135 (14.7)	0.281	163 (15.6)	161 (15.4)	0.795
	Agree	1297 (66.0)	1278 (65.0)		615 (66.8)	596 (64.7)		682 (65.3)	682 (65.3)	

Note: data presented as N (%). Strongly agree, agree and strongly disagree, disagree were combined as agree and disagree respectively. The McNemar test was used to obtain *p*-values.

**Table 4:** Odds ratios for dietary supplement use according to socio-demographics and COVID-19 status.

	Multivitamin		Vitamin D		Vitamin C		Zinc	
	OR (95%CI)	*p*	OR (95%CI)	*p*	OR (95%CI)	*p*	OR (95%CI)	*p*
Age (Years)								
15–25 Years	0.6 (0.5–0.8)	0.001	0.7 (0.5–1.0)	0.036	0.8 (0.5–1.2)	0.261	1.4 (0.5–4.2)	0.528
26–35 Years	0.9 (0.7–1.2)	0.475	0.9 (0.7–1.3)	0.623	1.2 (0.8–1.7)	0.386	0.7 (0.2–2.5)	0.541
36–45 Years	1.0 (0.7–1.3)	0.821	0.9 (0.7–1.3)	0.617	1.1 (0.7–1.6)	0.811	1.7 (0.5–5.2)	0.365
>45 Years	Reference							
Gender								
Female	0.9 (0.7–1.1)	0.329	0.9 (0.7–1.1)	0.334	1.0 (0.8–1.3)	0.967	0.9 (0.4–2.0)	0.893
Male	Reference							
Income (SAR)								
<5000 SAR	0.9 (0.7–1.2)	0.473	0.9 (0.7–1.2)	0.357	1.1 (0.8–1.6)	0.628	1.0 (0.4–2.5)	0.994
5000–7000 SAR	1.2 (0.8–1.7)	0.410	1.2 (0.8–1.7)	0.400	2.0 (1.3–3.2)	0.003	0.3 (0.0–2.5)	0.268
8000–16,000 SAR	1.1 (0.9–1.5)	0.337	1.0 (0.7–1.3)	0.929	1.5 (1.0–2.2)	0.028	0.7 (0.3–2.1)	0.578
>16,000 SAR	Reference							
Nationality								
Saudi	1.2 (0.9–1.6)	0.264	1.0 (0.8–1.4)	0.945	0.9 (0.6–1.3)	0.548	0.9 (0.3–2.4)	0.827
Non-Saudi	Reference							
Education								
High School	1.0 (0.7–1.6)	0.926	0.7 (0.4–1.1)	0.086	1.7 (1.0–3.0)	0.056	0.5 (0.1–2.3)	0.347
Bachelor	1.0 (0.7–1.3)	0.853	0.6 (0.5–0.9)	0.006	1.2 (0.8–1.8)	0.419	0.5 (0.2–1.3)	0.171
Master	1.2 (0.9–1.8)	0.249	0.7 (0.5–1.1)	0.108	1.2 (0.7–2.0)	0.455	0.2 (0.0–1.0)	0.052
PHD	Reference							
COVID-19 Status								
Positive	2.5 (1.4–4.5)	0.003	1.0 (0.5–2.0)	0.925	1.3 (0.6–2.9)	0.535	3.5 (0.8–15.4)	0.093
Negative	Reference							

Note: Data indicates odds ratios and 95% CI obtained using logistic regression with supplementation as dependent and socio-demographics as independent variables; *p* < 0.05 considered significant. Number of samples using Selenium were not enough to calculated ORs.

## Data Availability

The data used in this study is available on reasonable request from the corresponding author.

## Supplementary Materials

The following are available online at https://www.mdpi.com/article/10.3390/ijerph18126435/s1, Table S1: Percentage of supplement use in Gender and COVID-19 Patients and family members. Table S2: Percentage of supplement use according to COVID-19 status. Table S3: Gender distribution among participants for Supplementation use according to Education, Income, and Age. Table S4: Dietary Supplement use based on nationality and gender.
