# Supplementary material for: Dietary Intake and Supplement Use Among Saudi Residents during COVID-19 Lockdown
Source: Int J Environ Res Public Health. 2021 Jun 14;18(12):6435. doi: 10.3390/ijerph18126435 (PMC8296224; doi:10.3390/ijerph18126435)
Supplement: Supplementary file 1 [file ijerph-18-06435-s001.zip › ijerph-1245213-supplementary.pdf]

Table S1. Percentage of supplement use in Gender and COVID-19 Patients and family members.

Parameters	Overall			Covid 19 Patients and Family members		
	**Male**	**Female**	**P-values**	**Male**	**Female**	***P*-values**
Multivitamin						
No	682 (74.0)	793 (76.0)	0.329	10 (52.6)	15 (57.7)	0.736
Yes	239 (26.0)	251 (24.0)		9 (47.4)	11 (42.3)	
Vitamin D						
No	723 (78.5)	838 (80.3)	0.334	17 (89.5)	19 (73.1)	0.264
Yes	198 (21.5)	206 (19.7)		2 (10.5)	7 (26.9)	
Selenium						
No	915 (99.3)	1041 (99.7)	0.320	19 (100.0)	26 (100.0)	NA
Yes	6 (0.7)	3 (0.3)		0 (0.0)	0 (0.0)	
Vitamin C						
No	806 (87.5)	913 (87.5)	1.000	17 (89.5)	21 (80.8)	0.681
Yes	115 (12.5)	131 (12.5)		2 (10.5)	5 (19.2)	
Zinc						
No	908 (98.6)	1030 (98.7)	1.000	18 (94.7)	25 (96.2)	1.000
Yes	13 (1.4)	14 (1.3)		1 (5.3)	1 (3.8)	

Note: Data presented as N(%). P-values are obtained from chi-square test.

Table S2. Percentage of supplement use according to COVID-19 status.

Parameter	Are you or Your family member was diagnosed with COVID-19?			No COVID-19			COVID-19 Patients and family members		
	**NO**	**Yes**	**P-values**	**Male**	**Female**	**P-values**	**Male**	**Female**	***P*-values**
Multivitamin									
No	1450 (75.5)	25 (55.6)	0.003	672 (74.5)	778 (76.4)	0.328	10 (52.6)	15 (57.7)	0.736
Yes	470 (24.5)	20 (44.4)		230 (25.5)	240 (23.6)		9 (47.4)	11 (42.3)	
Vitamin D									
No	1525 (79.4)	36 (80.0)	0.551	706 (78.3)	819 (80.5)	0.238	17 (89.5)	19 (73.1)	0.264
Yes	395 (20.6)	9 (20.0)		196 (21.7)	199 (19.5)		2 (10.5)	7 (26.9)	
Selenium									
No	1911 (99.5)	45 (100.0)	1.000	896 (99.3)	1015 (99.7)	0.320	19 (100.0)	26 (100.0)	NA
Yes	9 (0.5)	0 (0.0)		6 (0.7)	3 (0.3)		0 (0.0)	0 (0.0)	
Vitamin C									
No	1681 (87.6)	38 (84.4)	0.533	789 (87.5)	892 (87.6)	0.921	17 (89.5)	21 (80.8)	0.681
Yes	239 (12.4)	7 (15.6)		113 (12.5)	126 (12.4)		2 (10.5)	5 (19.2)	
Zinc									
No	1895 (98.7)	43 (95.6)	0.126	890 (98.7)	1005 (98.7)	0.918	18 (94.7)	25 (96.2)	1.000
Yes	25 (1.3)	2 (4.4)		12 (1.3)	13 (1.3)		1 (5.3)	1 (3.8)	

Note: Data presented as N(%). P-values are obtained from chi-square test.

Table S3. Gender distribution among participants for Supplementation use according to Education, Income, and Age.

Parameters	Education											
	**High School**			**Bachelor**			**Master**			**PHD**		
	**Male**	**Female**	**P-value**	**Male**	**Female**	**P-value**	**Male**	**Female**	**P-value**	**Male**	**Female**	***P*-value**
Multivitamin												
No	52 (71.2)	71 (82.6)	0.089	408 (75.4)	452 (77.4)	0.434	110 (70.1)	151 (73.3)	0.497	102 (77.9)	104 (73.2)	0.375
Yes	21 (28.8)	15 (17.4)		133 (24.6)	132 (22.6)		47 (29.9)	55 (26.7)		29 (22.1)	38 (26.8)	
Vitamin D												
No	59 (80.8)	70 (81.4)	0.927	426 (78.7)	484 (82.9)	0.078	123 (78.3)	163 (79.1)	0.857	98 (74.8)	102 (71.8)	0.579
Yes	14 (19.2)	16 (18.6)		115 (21.3)	100 (17.1)		34 (21.7)	43 (20.9)		33 (25.2)	40 (28.2)	
Selenium												
No	71 (97.3)	85 (98.8)	0.594	537 (99.3)	583 (99.8)	0.201	157 (100.0)	205 (99.5)	1.000	131 (100.0)	142 (100.0)	NA
Yes	2 (2.7)	1 (1.2)		4 (0.7)	1 (0.2)		0 (0.0)	1 (0.5)		0 (0.0)	0 (0.0)	
Vitamin C												
No	62 (84.9)	70 (81.4)	0.554	475 (87.8)	513 (87.8)	0.983	136 (86.6)	181 (87.9)	0.725	116 (88.5)	128 (90.1)	0.67
Yes	11 (15.1)	16 (18.6)		66 (12.2)	71 (12.2)		21 (13.4)	25 (12.1)		15 (11.5)	14 (9.9)	
Zinc												
No	72 (98.6)	85 (98.8)	1.000	534 (98.7)	577 (98.8)	0.885	157 (100.0)	204 (99.0)	0.508	127 (96.9)	139 (97.9)	0.714
Yes	1 (1.4)	1 (1.2)		7 (1.3)	7 (1.2)		0 (0.0)	2 (1.0)		4 (3.1)	3 (2.1)	
	**Income**											
**Parameters**	**<5000**			**5000-7000**			**8000-16000**			**>16000**		
	**Male**	**Female**	**P-value**	**Male**	**Female**	**P-value**	**Male**	**Female**	**P-value**	**Male**	**Female**	**P-value**
Multivitamin												
No	250 (75.3)	273 (79.6)	0.182	68 (74.7)	76 (72.4)	0.711	182 (72.8)	233 (75.2)	0.526	172 (75.1)	196 (75.4)	0.944
Yes	82 (24.7)	70 (20.4)		23 (25.3)	29 (27.6)		68 (27.2)	77 (24.8)		57 (24.9)	64 (24.6)	
Vitamin D												
No	265 (79.8)	282 (82.2)	0.427	69 (75.8)	79 (75.2)	0.924	197 (78.8)	248 (80.0)	0.727	175 (76.4)	210 (80.8)	0.241
Yes	67 (20.2)	61 (17.8)		22 (24.2)	26 (24.8)		53 (21.2)	62 (20.0)		54 (23.6)	50 (19.2)	
Selenium												
No	330 (99.4)	343 (100.0)	0.242	91 (100.0)	103 (98.1)	0.500	246 (98.4)	309 (99.7)	0.178	229 (100.0)	260 (100.0)	NA
Yes	2 (0.6)	0 (0.0)		0 (0.0)	2 (1.9)		4 (1.6)	1 (0.3)		0 (0.0)	0 (0.0)	
Vitamin C												
No	298 (89.8)	305 (88.9)	0.724	77 (84.6)	83 (79.0)	0.315	211 (84.4)	266 (85.8)	0.641	203 (88.6)	238 (91.5)	0.283
Yes	34 (10.2)	38 (11.1)		14 (15.4)	22 (21.0)		39 (15.6)	44 (14.2)		26 (11.4)	22 (8.5)	
Zinc												
No	328 (98.8)	337 (98.3)	0.752	90 (98.9)	105 (100.0)	0.464	247 (98.8)	306 (98.7)	1.000	225 (98.3)	257 (98.8)	0.711

Yes	4 (1.2)	6 (1.7)		1 (1.1)	0 (0.0)		3 (1.2)	4 (1.3)		4 (1.7)	3 (1.2)	

**Parameters**	**Age**											
	**15-25 years**			**26-35 years**			**36-45 years**			**>45 years**		
	**Male**	**Female**	**P-value**	**Male**	**Female**	**P-value**	**Male**	**Female**	**P-value**	**Male**	**Female**	***P*-value**
Multivitamin												
No	224 (78.9)	254 (82.5)	0.268	160 (69.9)	216 (77.4)	0.054	136 (76.0)	156 (71.9)	0.358	152 (72.4)	152 (71.0)	0.757
Yes	60 (21.1)	54 (17.5)		69 (30.1)	63 (22.6)		43 (24.0)	61 (28.1)		58 (27.6)	62 (29.0)	
Vitamin D												
No	231 (81.3)	256 (83.1)	0.571	176 (76.9)	223 (79.9)	0.401	142 (79.3)	169 (77.9)	0.727	157 (74.8)	171 (79.9)	0.206
Yes	53 (18.7)	52 (16.9)		53 (23.1)	56 (20.1)		37 (20.7)	48 (22.1)		53 (25.2)	43 (20.1)	
Selenium												
No	283 (99.6)	308 (100.0)	0.480	227 (99.1)	277 (99.3)	1.000	176 (98.3)	217 (100.0)	0.092	210 (100.0)	213 (99.5)	1.000
Yes	1 (0.4)	0 (0.0)		2 (0.9)	2 (0.7)		3 (1.7)	0 (0.0)		0 (0.0)	1 (0.5)	
Vitamin C												
No	252 (88.7)	283 (91.9)	0.194	198 (86.5)	236 (84.6)	0.551	153 (85.5)	189 (87.1)	0.64	186 (88.6)	184 (86.0)	0.424
Yes	32 (11.3)	25 (8.1)		31 (13.5)	43 (15.4)		26 (14.5)	28 (12.9)		24 (11.4)	30 (14.0)	
Zinc												
No	280 (98.6)	303 (98.4)	1.000	228 (99.6)	276 (98.9)	0.631	176 (98.3)	213 (98.2)	1.000	206 (98.1)	213 (99.5)	0.212
Yes	4 (1.4)	5 (1.6)		1 (0.4)	3 (1.1)		3 (1.7)	4 (1.8)		4 (1.9)	1 (0.5)	

Note: Data presented as N(%) . P-values are obtained from chi-square test.

Table S4. Dietary Supplement use based on nationality and gender.

Parameters	Saudi			Non-Saudi		
	**Male**	**Female**	**P-value**	**Male**	**Female**	***P*-value**
Multivitamin						
No	568 (74.4)	627 (75.3)	0.704	104 (74.8)	151 (81.6)	0.139
Yes	195 (25.6)	206 (24.7)		35 (25.2)	34 (18.4)	
Vitamin D						
No	600 (78.6)	668 (80.2)	0.442	106 (76.3)	151 (81.6)	0.238
Yes	163 (21.4)	165 (19.8)		33 (23.7)	34 (18.4)	
Selenium						
No	760 (99.6)	831 (99.8)	0.675	136 (97.8)	184 (99.5)	0.318
Yes	3 (0.4)	2 (0.2)		3 (2.2)	1 (0.5)	
Vitamin C						
No	671 (87.9)	731 (87.8)	0.909	118 (84.9)	161 (87.0)	0.582
Yes	92 (12.1)	102 (12.2)		21 (15.1)	24 (13.0)	
Zinc						
No	754 (98.8)	821 (98.6)	0.648	136 (97.8)	184 (99.5)	0.318
Yes	9 (1.2)	12 (1.4)		3 (2.2)	1 (0.5)	

Note: Data presented as N(%); (%). P-values are obtained from chi-square test.
